# The Slack Channel Deletion Causes Mechanical Pain Hypersensitivity in Mice

**DOI:** 10.3389/fnmol.2022.811441

**Published:** 2022-03-11

**Authors:** Ye Liu, Fang-Fang Zhang, Ying Song, Ran Wang, Qi Zhang, Zhong-Shan Shen, Fei-Fei Zhang, Dan-Ya Zhong, Xiao-Hui Wang, Qing Guo, Qiong-Yao Tang, Zhe Zhang

**Affiliations:** ^1^Jiangsu Province Key Laboratory of Anesthesiology, Xuzhou Medical University, Xuzhou, China; ^2^Jiangsu Province Key Laboratory of Anesthesia and Analgesia Application Technology, Xuzhou Medical University, Xuzhou, China; ^3^NMPA Key Laboratory for Research and Evaluation of Narcotic and Psychotropic Drugs, Xuzhou Medical University, Xuzhou, China

**Keywords:** Slack channel, SOM^+^ neurons, DRG neurons, mechanical pain, potassium channel

## Abstract

The role of the Slack (also known as Slo2.2, K_Na_1.1, or KCNT1) channel in pain-sensing is still in debate on which kind of pain it regulates. In the present study, we found that the Slack^–/–^ mice exhibited decreased mechanical pain threshold but normal heat and cold pain sensitivity. Subsequently, X-gal staining, *in situ* hybridization, and immunofluorescence staining revealed high expression of the Slack channel in Isolectin B4 positive (IB4^+^) neurons in the dorsal root ganglion (DRG) and somatostatin-positive (SOM^+^) neurons in the spinal cord. Patch-clamp recordings indicated the firing frequency was increased in both small neurons in DRG and spinal SOM^+^ neurons in the Slack^–/–^ mice whereas no obvious slow afterhyperpolarization was observed in both WT mice and Slack^–/–^ mice. Furthermore, we found *Kcnt1* gene expression in spinal SOM^+^ neurons in Slack^–/–^ mice partially relieved the mechanical pain hypersensitivity of Slack^–/–^ mice and decreased AP firing rates of the spinal SOM^+^ neurons. Finally, deletion of the Slack channel in spinal SOM^+^ neurons is sufficient to result in mechanical pain hypersensitivity in mice. In summary, our results suggest the important role of the Slack channel in the regulation of mechanical pain-sensing both in small neurons in DRG and SOM^+^ neurons in the spinal dorsal horn.

## Introduction

The Slack channel (Slo2.2, K_Na_1.1) (Kaczmarek et al., 2017), encoded by the *Kcnt1* gene, is a Na^+^-activated potassium channel belonging to the Slo channel family (Alex Yuan et al., 2003; Salkoff et al., 2006). The α-subunit contains six transmembrane segments, a cytoplasmic N-terminal and a long C-terminal domain (William J. Joiner et al., 1998). The C-terminus possesses two regulators of K^+^ conductance (RCK) domains, essential for ligand binding and concomitant channel gating (Zhang et al., 2010). The Slack channel modulates slow afterhyperpolarization (s-AHP) following repetitive action potentials (APs) (Bhattacharjee and Kaczmarek, 2005; Wallen et al., 2007; Brown et al., 2008; Gao et al., 2008) and is associated with epilepsy (Heron et al., 2012; Ohba et al., 2015; Arai-Ichinoi et al., 2016), ALS (Zhang et al., 2017), and Fragile X syndrome (Noebels, 2015; Ferron, 2016). The Slack channel is richly expressed in the brain and dorsal root ganglia (DRG) (Bhattacharjee et al., 2002; Tamsett et al., 2009; Lu et al., 2015) suggesting its involvement in pain sensing. The Slack channel is also reported expressed in IB4-positive central terminals in the spinal cord (Lu et al., 2015; Lu et al., 2021).

The earliest study on the role of the Slack channel in pain proposed that intrathecal injection of *Kcnt1* siRNA could enhance thermal pain sensitivity at 50°C and mechanical pain sensitivity (Huang et al., 2013). However, a subsequent study stated that spared nerve injury (SNI) induced mechanical hypersensitivity was significantly increased in Slack channel null mice compared with SNI control mice. But there is no alteration in basal pain and inflammation pain in naïve Slack null mice (Lu et al., 2015). In another line of Slack channel knockout mice, the Slack channel was reportedly involved in the itching sensation but not in thermal pain sensitivity (Martinez-Espinosa et al., 2015). However, recent studies have reported that Slack channel deletion altered nociceptive responsiveness to thermal stimuli by altering neuron excitability in the spinal dorsal horn (Evely et al., 2017). Thus, previous results are inconsistent and further investigations are required to disclose the role of the Slack channel in regulating pain.

In the present study, we examined pain behavior using a new line of Slack channel knockout (Slack^–/–^) mice, in which the Exon 7–11 in the KCNT1 gene was replaced by IRES-lacZ-cassette. We found that Slack^–/–^ mice were hypersensitive to mechanical stimuli but maintained normal responses to thermal and cold stimuli. Further experiments revealed the expression pattern of the Slack channel in the DRG and spinal cord and characterized the electrophysiological properties of neurons in the DRG and somatostatin (SOM^+^) neurons in the spinal cord of WT and Slack^–/–^ mice. Meanwhile, *Kcnt1* gene expression in spinal SOM^+^ neurons in Slack^–/–^ mice reversed their hypersensitivity to nociceptive mechanical stimuli.

## Materials and Methods

### Animals

**FIGURE 1:**
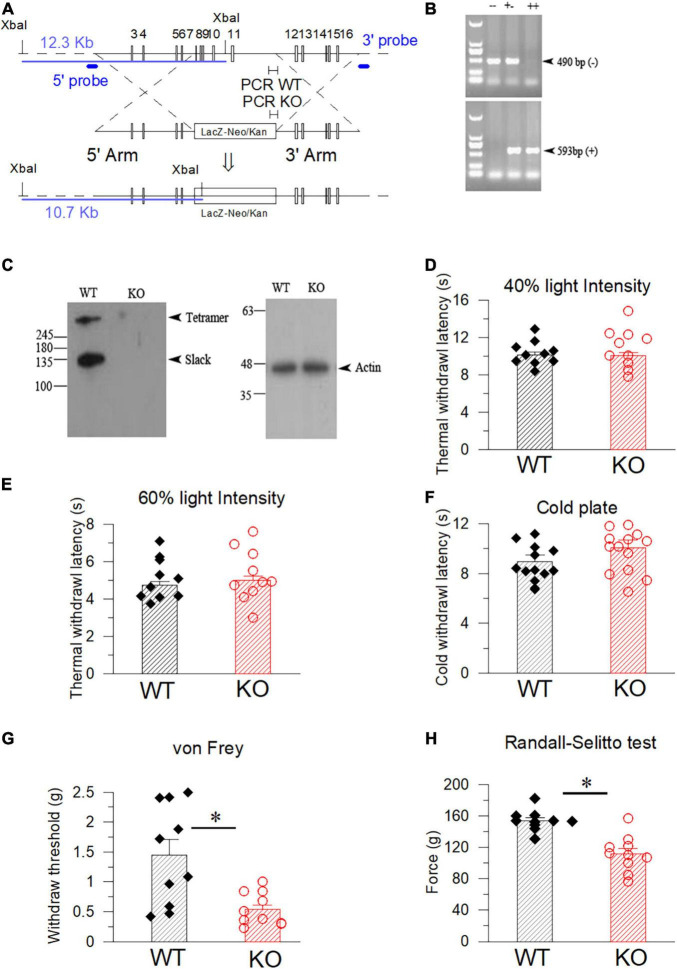
The strategy of constructing and validating the Slack^–/–^ mice and examination of its pain behavior. **(A)** The KCNT1 targeting construct in which the DNA sequence of genome locus containing 7–11 exon of the Slack channel gene was replaced by IRES-Lacz-Neo/Kan Cassette. The nuclease cleavage site, the Southern Blot probe, and expected bands of the WT and Slack^–/–^ mice were shown. The locus of the PCR fragment for identification of the WT mice and Slack KO mice at the indicated position. **(B)** PCR verification of the mice genotypes using the mice tail tissue. The PCR product shows a band of 593bp in electrophoresis for WT mice, while the amplicon for the Slack^–/–^ mice is 490 bp. PCR products of the heterozygote mice contain both fragments. **(C)** Western Blot verification of missing the Slack channel protein using the protein samples extracted from the spinal cord tissues of the WT and Slack^–/–^ mice. **(D,E)** WT mice and Slack^–/–^ mice exhibit no difference in paw withdrawal time under light-induced hot planar test with 40% light intensity (WT: 10.2 ± 0.3 s, *n* = 10; Slack^–/–^: 10.1 ± 0.3 s, *n* = 10. Student *T*-test: *P* = 0.49) and 60% light intensity (WT: 4.8 ± 0.2 s, *n* = 10; Slack^–/–^: 5.0 ± 0.2 s, *n* = 10; *P* = 0.74). **(F)** WT mice and Slack^–/–^ mice exhibit no difference in paw withdrawal time under dry ice induced cold planar test (WT: 9 ± 0.5 s, *n* = 13, Slack^–/–^: 10 ± 0.6 s, *n* = 13; *P* = 0.67). **(G)** Mechanical thresholds of the WT mice (1.4 ± 0.3 g, *n* = 10) and Slack^–/–^ mice (0.5 ± 0.1 g, *n* = 10) show a statistically significant difference (von Frey test, student *T*-test: *P* = 0.035, *indicates *P* < 0.05). **(H)** Enhanced pain threshold in Slack^–/–^ mice with Randall-Selitto test (WT: 154 ± 4.2 g, *n* = 10; Slack^–/–^: 112 ± 6.9 g, *n* = 10; student *T*-test: *P* = 0.0004, *indicates *P* < 0.05).

Slack^–/–^ mice were purchased from Deltagen incorporation (United States) and were maintained in Charles River incorporation before shipping to us. Slack^–/–^ mice were generated by homologous recombination using a *Slack-KO* targeting vector. The vector possesses a 5′ homology arm containing exons 3–6 and a 3′ homology arm containing exons 12–16. The *Slack-KO* targeting vector also contains an IRES-LacZ-NEO/Kan cassette, which replaced the genome DNA sequence encoding exons 7 through 11 that encodes the Slack channel pore domain. Slack^–/–^ mice (C57/B6) were backcrossed to the C57BL/6J background for > 10 generations (Figure 1A). Genotyping was routinely performed by PCR on genomic DNA isolated from tail-tip biopsies using specific primer sets as follows: WT 5′ primer GGGTGGGATTAGATAAATGCCTGCTCT, Slack KO 5′ primer ACTATTGCTTTATGATAATGTTTCATAG, common 3′ primer: GGAGGCTGCCACAACCATGATACC. Western Blot detected KCNT1 channel protein expression in the spinal cord tissues of the WT littermates, but not in Slack^–/–^ mice (Figure 1C). SOM^+^-Cre mice and Ai9 mice were gifts from Professor Yangang Sun from the Institute of Neuroscience in Shanghai. SOM^+^-Cre mice were mated with Ai9 (Rosa26-loxP-STOP-loxP-tdTomato) mice to generate SOM^+^-Tomato^+^ mice, in which red fluorescence was used to label SOM^+^ neurons. SOM^+^-tomato^+^ mice were then mated with Slack^–/–^ mice to generate SOM^+^-tomato^+^-Slack^+/–^ mice, then the SOM^+^-tomato^+^-Slack^+/–^ were crossed with the SOM^+^-tomato^+^-Slack^+/–^ mice. They produced offspring were selected to generate SOM^+^-tomato^+^-Slack^–/–^ mice and SOM^+^-tomato^+^-Slack^+/+^ mice. All experiments related to mouse generation and subsequent operation and protocols were approved by the Institutional Animal Care and Use Committee (IACUC) of Xuzhou Medical University. All mice were bred at the core facility of Xuzhou Medical University on a 12/12 h light/dark cycle with free access to food and water.

The Loxp sites flanked Slack CKO mice were purchased from GemPharmatech Co. Ltd., Nanjing, China. Genotyping primers: 5′ Arm primer: JS04692-Kcnt1-5wt-tF1, GTACCTCACTCCCTGCTCTGACAG, JS04692-Kcnt1-5wt-tR 1, GCAGTCTGTGTTGAGTGTTGACTGC, 3′ Arm primer: JS04692-Kcnt1-3wt-tF1, CAGATGGGCAGTGACAGACAGAC A, JS04692-Kcnt1-3wt-tR1, AAAGAGATGCCTTGGAGCATGG. The CKO mice were realized by intrathecal injection of rAAV-fSST-Cre-bGHpA (Brain VTA, Wuhan, China) with a titration concentration of 5E+12 VG/ml. One mouse was injected with 1 μl virus diluted in 4 μl normal saline twice at 24 h intervals. The mice in the control group were injected rAAV-fSST-EGFP-bGHpA (Brain VTA, Wuhan, China) with the same amount.

### Immunoblot for Detecting Slack Channel Protein Expression in the Spinal Cord of WT Mice, Slack^–/–^ and WT Mice With Compensated Slack Channel Expression

Total protein was extracted from homogenized spinal cord tissues of the WT, Slack^–/–^ and Slack^–/–^ mice with compensated Slack channel expression by ITH using a kit according to the manufacturer’s protocol (KeyGEN). For comparing the protein expression level of the Slack channel in WT mice with the Slack channel CKO mice in the spinal cord, the total protein was extracted from lumbar sections (L4, L5) of the spinal dorsal horn. The spinal cord of WT and Slack CKO mice were transversely cut to obtain the L4, 5 spinal sections, subsequently the sections were further coronal cut in a frozen state to remove the spinal ventral horn. The tissues were homogenized in lysate solution with a protease inhibitor cocktail and transferred into a 1.5 ml Eppendorf tube. The lysates were centrifuged at 12,000 rpm for 10 min at 4°C, supernatants were collected. Protein concentration and yield were determined using a BCA protein assay kit (Beyotime). Cytosolic protein (200 μg) was electrophoresed and transferred by SDS-PAGE using 12.5% (w/v) acrylamide gels and blotted onto PVDF membranes (Immobilon-P). Protein blots were blocked with 5% non-fat milk in TBST. An appropriate primary antibody (mouse anti-slo2.2, 1:1000; NeuroMab, clone N3/26, or anti-FLAG tag) was added and incubated overnight at 4°C. After washing, the membrane was incubated for another 1 h at room temperature with a secondary antibody: HRP goat anti-mouse IgG (H+L) (1:2000, ABclonal). The membrane was washed again in TBST, developed with a clear western ECL substrate (Beyotime), and imaged on an Alliance Q9 Imager (Uvitec). Band intensities were determined using the ImageJ software.

### Pain Behavioral Testing

Male mice (12–15 w) were used for all behavioral studies. The animals were habituated to the experimental room for 1 h to adapt to the environment.

#### Randall-Selitto Test

Nociceptive withdrawal thresholds to noxious mechanical stimuli were determined using a Randall-Selitto digital pressure instrument (IITC Life Science, Italy). Each mouse received 5 min of handling for manipulation before the test. Subsequently, the tested paw was gently placed onto the equipment platform and the mouse was kept in a quiet, comfortable environment. The device tip was applied to the medial plantar surface of the hind paws. The pressure on the plantar surface of the hind paw gradually increased until an escape response was elicited. The terminal pressure at the moment of escape response was recorded. Each mouse was tested three times at 15-min intervals.

#### Hargreaves Test

The Hargreaves test was performed using BME-410A type fully automatic heat-pain stimulators (K Hargreaves et al., 1988). Mice were placed on a 3 mm thick glass plate in small square air-permeable boxes for 1 h to adapt to the environment. An infrared radiation stimulator was positioned underneath the mice and irradiated at the plantar surface of the hind paw. The time needed to withdraw from the heat stimulus was automatically recorded as withdrawal latency. The cut-off time set in the Hargreaves testing is 25 s. Each mouse was tested three times at 15-min intervals.

#### Cold Plate Test

Mice were placed on a 2 mm thick glass plate with small square air-permeable boxes for 1 h. A syringe without the tip was fused with dry ice, placed underneath the mouse’s hind paw, touching the glass plate. The withdrawal time from the cold stimulus was recorded as withdrawal latency. The experiment started at 25–26°C. The average withdrawal temperature is 22–24°C. Each mouse was tested three times at 15-min intervals (Brenner et al., 2012).

#### Von Frey Test

Mice were placed individually in small cages with a penetrable bottom. A monofilament was applied perpendicularly to the plantar surface of the hind paw until it buckled, delivering a constant predetermined force (0.008–6 g) for 5 s. A positive response was recorded if the mice exhibited paw withdrawal, either during stimulus application or immediately after the filament was removed. When a positive signal was observed, the filament was shifted to a filament with a smaller force next to the filament to produce a positive response. If no positive signal was observed, the filament was shifted to a filament with a larger force next to the one that was used. The calculation and statistical analysis were performed according to a previously described protocol (Minett et al., 2011; Huang et al., 2020).

### X-Gal Staining, Immunohistochemistry, and *in situ* Hybridization

#### Section Preparation

Mice were anesthetized with 1% pentobarbital (vol/weight, i.p.) and immediately intracardially perfused through a natural vascular network with 0.9% saline, followed by 4% paraformaldehyde (4% PFA-DEPC for *in situ* hybridization) in PBS (pH 7.4). The lumbar spinal cord (L4-L5) and DRGs (L4-L5) were dissected and placed in 4% fresh PFA overnight at 4°C for further fixation. The tissues were then dehydrated in a gradient sucrose solution from 10 to 30% (30% sucrose-DEPC for *in situ* hybridization). Subsequently, tissues were frozen and buried in OCT gel to prepare cryostat sections in a cryo-cut microtome (LEICA, CM1950). The sections were cut to a thickness of 20 μm (15 μm for *in situ* hybridization) thickness and stored at −80°C.

#### X-Gal Staining

The sections obtained from Slack knockout mice were fixed in fixation solution and then placed in freshly prepared 0.1% X-gal solution for overnight incubation (12–16 h). After blue staining appeared, the sections were placed in wash buffer (195.6 ml 0.1 M PB, 0.4 ml 1 M MgCl_2_, 2 ml 2% NP-40) for washing. Before sealing the coverslips, the slices were dehydrated with ethanol and made transparent using xylene.

#### Double Immunofluorescence Staining and Image Taken

Sections were permeabilized with PBST (0.1% Triton X-100 in PBS) for 5 min, blocked with 10% normal goat serum in PBS for 60 min, and incubated with primary antibodies diluted in 3% BSA in PBS (containing 1 mM CaCl_2_ 1 mM MgCl_2_, 1 mM MnCl_2_, and 0.2% Triton X-100, pH 7.4; Sangon) overnight at 4°C. The following antibodies were used: mouse anti-slo2.2 (1:400; NeuroMab, clone N3/26), rabbit anti-calcitonin gene-related peptide (anti-CGRP; 1:800; Calbiochem), rabbit anti-NF200 (1:1000; Sigma-Aldrich, United States), rabbit anti-SOM (1:200, Thermo Fisher, United States), and rabbit anti-Dynorphin (1:200, Thermo Fisher, United States). Subsequently, sections were incubated at room temperature for 30 min and washed with PBS three times for 5 min each time. The cells were then stained with secondary antibodies conjugated with Alexa Fluor 488 (1:200, Life Technologies, United States) or 594 (1:200, Life Technologies, United States). For staining with isolectin B4 (IB4), sections were incubated with Alexa Fluor 488-conjugated IB4 (10 μg/ml in PBS buffer, Invitrogen) for 2 h at room temperature. After incubation, sections were washed three times in PBS for 5 min and sealed with coverslips after adding the quenching-preventive agent.

Images were acquired and preserved using an Olympus confocal microscope (Olympus, FV1000). Control slices were stained by omitting the first antibodies and incubating the tissues of Slack^–/–^ mice. The double staining cells, dynorphin, or somatostatin positive neurons were manually counted by randomly selecting 5 square fields from lamina I to V of the dorsal horn on each section. 10–12 hemisections at the lumbar from 3 mice were counted.

#### *In situ* Hybridization

On the first day, slices were rewarmed at room temperature and, fixed in 4% PFA-DEPC for 20 min, treated with PK-buffer (50 mM Tris-HCl, 5 mM M EDTA, 5% Protease K) for 15 min. Subsequently, sections were treated with acetyl anhydride buffer to reduce background (1.3% triethanolamine, 20 mM HCl, 0.25% acetyl anhydride) for 10 min before adding hybridization buffer (50% formamide, 5 × SSC, 50 μg/ml yeast tRNA, 100 μg/ml heparin, 1× Denhardt buffer, 0.1% Tween20, 1% CHAPS, 5 mM EDTA) was added to the slices and placed in an oven (BOXUN, GZX-9070MBE) at 65°C for 2.5 h. Finally, the prepared probes were dropped on the brain slices for hybridization with targeted mRNA at 65°C overnight. DEPC-PBS was used as a washing buffer. On the second day, slices were washed with 0.2 × SSC for 15 min, 30 min, and 30 min, followed by treatment with PBST for 20 min. After incubation with goat serum for 2 h, an anti-digoxin antibody (1:2000) was added to the slices overnight. On the third day, the slices were first washed with PBST solution three times for 30 min each. This was followed by washing with AP buffer (100 mM Tris, 50 mM MgCl_2_, 100 mM NaCl, 0.1% Tween-20) twice for 15 min each. Next, the slides were stained in 200 ml AP Buffer with 0.5 uL NBT and 3.5 uL BCIP coloration, left in the dark for 2 h. Finally, sections were washed twice with PBS and fixed with 4% PFA for 15 min before coverslip coating.

### *In vitro* Patch-Clamp Recordings From Whole-Mount Dorsal Root Ganglions

L4 and L5 DRGs were removed carefully. After the connective tissue was cleaned, DRGs were digested with a mixture of 0.4 mg/mL trypsin (Sigma) and 1.0 mg/ml type-A collagenase (Sigma) for 45 min at 37°C. Then, they were transferred into ACSF (in mM: 124 NaCl, 2.5 KCl, 1.2 NaH_2_PO_4_, 1.0 MgCl_2_, 2.0 CaCl_2_, 25 NaHCO_3_, and 10 Glucose) to incubate at 28°C for ≥1 h and agitated by gentle bubbling with 95% O_2_ and 5% CO_2_. Next, the preparation was moved to a recording chamber. The entire ganglion was stabilized using a slice anchor. DRG neurons were visualized with a 40 × water-immersion objective using a microscope (BX51WI; Olympus, Tokyo, Japan) equipped with infrared differential interference contrast optics. Whole-cell voltage-clamp recordings were performed by using a Multiclamp 700 B amplifier (Molecular Devices Corporation, Sunnyvale, CA) after a gigaohm seal was established. The electrode had a final resistance of 4–7 MΩ and was filled with a normal pipette solution containing (in mmol/L) 120 potassium gluconate, 18 KCl, 2 MgCl_2_, 5 EGTA, 10 HEPES, 5 Na_2_-ATP, 0.4 Na-GTP, and 1 CaCl_2_ (pH 7.2 adjusted with KOH, osmolarity 300 mOsm). The series resistance was 10–20 MΩ. The pipette offset current was zeroed immediately before contacting the cell membrane, and the electrode capacitance was canceled after seal formation. Before electrode penetration, the DRG soma was visually classified according to its soma diameter as large (≥40 μm), middle size (26–40 μm), and small diameter (≤26 μm) neurons (Breese et al., 2005; Stemkowski et al., 2015). After establishing the whole-cell recording mode, series resistance was compensated to 70–75%. All chemicals were obtained from Sigma-Aldrich. Data were acquired with a Digidata 1322A acquisition system (molecular devices) using pCLAMP 9.0. Signals were low-pass filtered at 5 kHz, sampled at 10 kHz, and analyzed offline.

### Spinal Cord Slice Preparation and *in vitro* Patch-Clamp Recordings From Spinal Cord SOM^+^ Neurons

Mice were deeply anesthetized with sevoflurane, decapitated and lumbar spinal cord slices quickly removed to ice-cold modified cutting solution contain (in mM, titrated to pH 7.4): 85 NaCl, 2.5 KCl, 1.25 NaH_2_PO_4_, 4 MgCl_2_, 0.5 CaCl_2_, 24 NaHCO_3_, 25 Glucose, 75 Sucrose. The solution osmolarity was 300–320 mOsm and it was oxygenated with 95% O_2_ and 5% CO_2_. Transverse spinal cord slices (from L3–L5 segments, 240 μm) were obtained using a vibrating blade microtome (Leica VT-1000S, Heidelberg, Germany). Slices were incubated for approximately 1 h at 32°C in cutting solution and 15–30 min in ACSF (in mM: 126 NaCl, 2.5 KCl, 1.2 NaH_2_PO_4_, 1.2 MgSO_4_, 2.4 CaCl_2_, 26 NaHCO_3_, 10 Glucose with at pH 7.4, osmolarity at 290–310 mOsm) at room temperature, oxygenated with 95% O_2_ and 5% CO_2_.

Whole-cell patch-clamp recordings were performed at room temperature in dorsal horn SOM^+^ neurons visually identified using the infrared gradient contrast technique (Duan et al., 2014; Punnakkal et al., 2014). Slices were transferred to a recording chamber and continually perfused (4 ml/min) with an incubation chamber containing oxygenated ACSF, constantly bubbled with Carbonox (95% O_2_ and 5% CO_2_) to achieve a final pH of 7.3–7.4. The recordings were limited to neurons located within the superficial area of the spinal cord at room temperature. This area is easily identified under brightfield illumination by its translucent appearance in spinal cord slices and contains a discernible concentration of SOM^+^ neurons. Patch pipettes (6–8 MΩ) were pulled on a micropipette puller (P-97, Sutter Instrument, Novato, CA) and filled with a pipette solution containing (in mM): 130 potassium gluconate, 5 KCl, 4 Na_2_ATP, 0.5 NaGTP, 20 HEPES, 0.5 EGTA pH 7.3 or 7.4 (with KOH). APs were recorded in the current-clamp recording mode. The membrane potential recorded (I = 0) after switching from voltage to current clamp was designated as the RMP. AP firing was elicited by injecting a series of depolarizing step currents (2 s duration, 10 pA increments, form −60 to ∼180 pA) into the recorded neuron with the Sutter Patch system (Sutter, Igor Pro 8.02). The liquid junction potential was measured as 19 mV and was corrected by typing the value into the square box next to the right of the Auto Offset button in the Sutter software. Once the whole-cell configuration formation, the input resistance is stabled at several hundred MΩ were accepted as good patches to keep recording. The Rs were monitored during recording, the patches with Rs alteration of more than 15% were discarded. Rs compensation was turned on and set to 85%. The AP amplitude was defined as the distance from the baseline to the peak of AP. AP-half width was quantified as AP duration measured at half of the maximum amplitude relative to the resting membrane potential. Tonic firing cells were defined as AP discharge throughout the 500 ms current injections. Delayed firing neurons were defined as a delayed onset to spike during the depolarization phase after the start of a depolarizing current injection. Neurons with an AP latency above 30 ms upon injection of 100 pA current were clarified as the delayed firing neurons.

The sAHP was observed following an 80 pA current injection that last 1s train to generate multiple action potentials while the fAHP was observed following a 50 pA current injection that last 10 ms to generate only one action potential. The amplitude of fAHP or sAHP was calculated as the absolute value between the resting membrane potential and maximal negative amplitude after the train of firing at the end of the 80 pA (1 s) depolarizing current injection.

s-EPSC were studied in voltage-clamp recording mode at a holding potential of −70 mV (Smith et al., 2015) for 2 min with SutterPatch (Sutter, Igor Pro 8.02). AP firing was recorded after ITH using the Patchmaster system (HEKA, EPC10) with step-currents (1 s duration, 10 pA increments, −60 to ∼150 pA). The sEPSC were analyzed using the Sutter Igor Pro software with the synaptic events analysis function. After the template was set, the synaptic events were automatically picked by the software but with manual selection. The decay time course was fitted with exponential decay function *F*(*t*) = *B* + *Ae*^−(*t*−*t*_0_)/*t*_τ_^. The activation time course was fitted by using the function $Im\left(t\right)=B+A/\left(1-e^{-\frac{t-t_{0}}{t_{\tau }}}\right)$, where B is the baseline offset, A is the amplitude of the sEPSC, t_o_ is the time at which the sEPSC reached 50% of the full amplitude and τ gave a measure of the rate of rising from 10 to 90% rising time (Taylor et al., 1995). The amplitude of the sEPSC was calculated as the peak current minus the template current.

The Na^+^-dependent current recording was performed in the whole-cell recording configuration, and the net difference in current was observed from −80 mV to −120 mV in voltage-clamp recording mode. Na^+^-independent “leak” current was recorded with 0 Na^+^ (in mM, 0 NaCl, 140 KCl, 1 MgCl_2_, 5 EGTA, 10 HEPES, 3 Mg-ATP, 0.3 Na-GTP, pH 7.3 adjusted with KOH, osmolarity at 290–310 mOsm). Na^+^- dependent currents were observed with a 70 mM cytosolic Na^+^ (70 NaCl, 73.3 KCl, 1 MgCl_2_, 5 EGTA, 10 HEPES, 3 Mg-ATP, 0.3 Na-GTP, pH 7.3 adjusted with KOH, OsM 290–300) in the pipette solution. We used 20 mM CsCl in the ACSF (126 NaCl, 2.5 KCl, 1.2 NaH_2_PO_4_, 1.2 MgSO_4_, 2.4 CaCl_2_, 26 NaHCO_3_, 10 Glucose with pH at 7.4, osmolarity at 290–310 mOsm) as the K^+^ current inhibitor solution.

### Compensated Slack Channel Expression in the Spinal Cord by AAV Virus ITH

Mice were anesthetized with sevoflurane until the righting reflex disappeared. The spinous process of L6, which was the prominent one, was located, and the vertebrate column was gently fixed around this area. The syringe needle was carefully inserted between the groove of the L4 and L5 vertebrae, and a tail-flick was observed as a sign of successful entry of the needle into the intradural space. Once the tail-flick was observed, 5 μl virus was injected (Njoo et al., 2014). The virus carried the Slack channel was generated by Taitool Incorporation in Shanghai. The full length of Flag-tagged rKCNT1 was inserted into the AAV vector to generate the AAV2/9-hSyn-DIO-rKcnt1-3Flag-SV40-pA virus. The virus was intrathecal injected with a titer concentration of 1 × 10^12^. The control virus was AAV2/9-hSyn-DIO-mGFP-WPRE-pA and with a titer concentration of 1 × 10^12^ (Taitool, Shanghai), which guaranteed infection in the spinal cord but not in the DRG. Twenty-one days after intrathecal injection, the mice were used to examine the pain behavior. After behavior testing, the mice were sacrificed by CO_2_, and the DRG and spinal cord of mice were dissected and cryostat sectioned at a thickness of 20 μm. Successful GFP expression was observed using a laser scanning confocal microscope (ZEISS, LSM880, Germany), while Slack channel expression was confirmed by western blotting and electrophysiology.

### Statistical Analysis

Statistical analyses were performed with Sigma plot 14 software using the appropriate method illustrated in figure legends. All two-group comparison behavior data were analyzed using an unpaired *T*-test. A two-tail test method was used and the statistical results showed as mean ± SEM. A one-way ANOVA test was used for three or more groups comparisons. Data are considered to be statistically significant if *p* < 0.05.

## Results

### Slack^–/–^ Mice Are Hypersensitive to Mechanical Pain

To explore the relationships between the Slack channel and nociceptive pain, Slack^–/–^ mice were generated using the homologous recombination strategy (Deltagen incorporation, United States). In brief, a LacZ gene fragment box was inserted to replace exons 7–11 of the murine *Kcnt1* gene, which encodes the pore region of the Slack channel (Figure 1A). Homogenous Slack^–/–^ mice were identified by PCR using the chromosome DNA extracted from the mice’s tail as a template (Figure 1B). The Slack channel protein was not detected in total proteins extracted from the spinal cord of the Slack^–/–^ mice (Figure 1C). We then examined the pain behavior of Slack^–/–^ mice. Thermal pain sensitivity was examined using the Hargreaves test, finding no significant difference in paw withdrawal time between male WT and Slack^–/–^ mice either with 40 or 60% infrared light intensity (Figures 1D,E), indicating that Slack channel deletion did not alter their thermal pain sensitivity. Subsequently, cold pain sensitivity was measured using a cold plantar assay (Brenner et al., 2012). Male WT and Slack^–/–^ mice exhibited similar paw withdrawal latency, indicating unaltered cold pain sensitivity in Slack^–/–^ mice (Figure 1F). However, the paw withdrawal pressure forces in von Frey and Randall-Sellito tests in Slack^–/–^ mice were much lower than those needed in WT mice, indicating an enhanced mechanical pain sensitivity in male Slack^–/–^ mice (Figures 1G,H). All these tests were repeated using female Slack^–/–^ mice and got similar results (Supplementary Figure 1). These results suggest that the Slack channel plays a vital role in mechanical pain sensing rather than thermal and cold pain sensing.

### The Slack Channel Is Richly Expressed in IB4^+^ Neurons in the DRG and SOM^+^ Neurons in the Spinal Dorsal Horn

**FIGURE 2:**
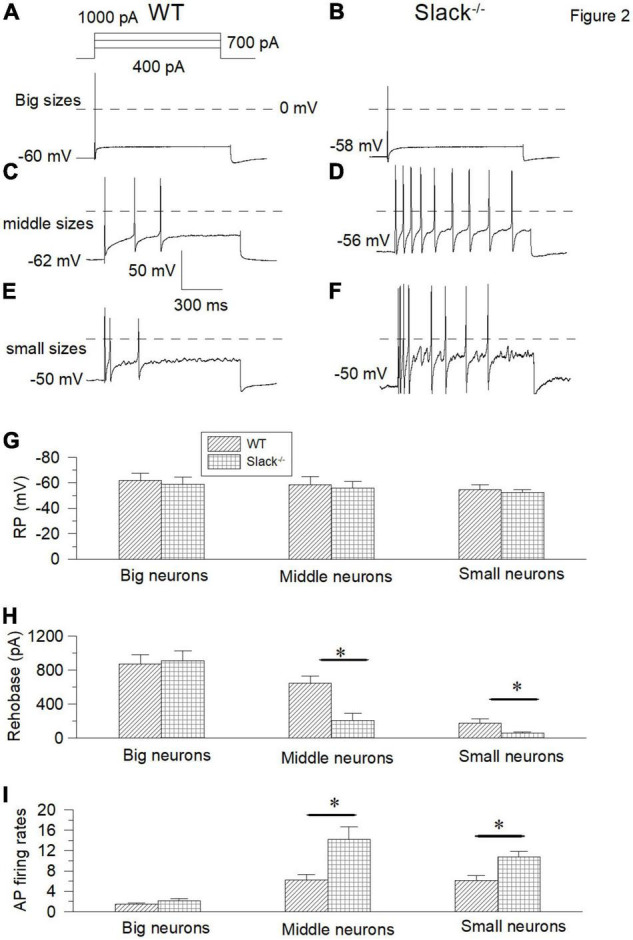
The small and middle-sized DRG neurons in the Slack^–/–^ mice exhibit overactivity. **(A,B)** Sample action potential traces of the big-sized DRG neurons in the WT and Slack^–/–^ mice were elicited by 1000 pA current injection. **(C,D)** The action potential traces of the middle-sized DRG neurons were elicited by 700 pA current injection. **(E,F)** Sample action potential traces of small-sized DRG neurons in the WT and Slack^–/–^ mice were elicited by 400 pA current injection. **(G)** Comparison of resting membrane potentials of the big-sized (WT, *n* = 10, Slack^–/–^, *n* = 10, Student *T*-test, *P* = 0.51), middle-sized (WT, *n* = 11, Slack^–/–^, *n* = 11, P = 0.64) and small-sized (WT, *n* = 11, Slack^–/–^, *n* = 11; *P* = 0.7) DRG neurons in the WT mice and Slack^–/–^ mice, respectively. **(H)** Comparison of rheobase currents in the big-sized (WT, *n* = 11; Slack^–/–^, *n* = 11; *P* = 0.63), middle-sized (WT, *n* = 11; Slack^–/–^, *n* = 11; *P* = 0.01) and small-sized (WT, *n* = 11; Slack^–/–^, *n* = 11; *P* = 0.005) DRG neurons in the WT mice and Slack^–/–^ mice, respectively. **(I)** Comparison of action potential firing rates of the big-sized (WT, *n* = 8; Slack^–/–^, *n* = 8; *P* = 0.61), middle-sized (WT, *n* = 10; Slack^–/–^, *n* = 10; *P* = 0.014) and small-sized (WT, *n* = 12; Slack^–/–^, *n* = 12; *P* = 0.012) DRG neurons in the WT mice and Slack^–/–^ mice, respectively. *P < 0.05.

To address the mechanism of mechanical hyperalgesia in Slack^–/–^ mice, we measured the expression levels of the Slack channel in the DRG and spinal cord. Since the LacZ gene, which encodes the β-galactosidase, was under the control of regulatory elements corresponding to the *Kcnt1* gene in Slack^–/–^ mice, we first performed X-gal staining to observe the Slack channel distribution in the spinal dorsal horn and DRG. We detected indigo staining signals in both the DRG and spinal cord in Slack^–/–^ mice (Supplementary Figures 2A,C), but not in WT mice (Supplementary Figures 2B,D). Slack channel expression is rich in the spinal dorsal horn, but with a scattered expression pattern in other parts of the spinal cord. This expression pattern was further confirmed by *in situ* hybridization in WT mice (Supplementary Figures 2E,F). The expression of Slack channel in the spinal dorsal horn in WT mice was further confirmed by immunofluorescence staining (Supplementary Figures 2G,H). To further clarify the category type of neurons where the Slack channel is expressed in the DRG and Spinal cord, double immunofluorescence staining was performed. In DRG neurons, the absence of co-localization of the Slack channel (in red) and NF200 (in green) indicates that the Slack channel is scarcely expressed in large-diameter neurons (Supplementary Figures 3A–D). Meanwhile, the Slack channel (in red) is only expressed in a small amount (28 ± 2.4%) of CGRP^+^ neurons (in green) (Supplementary Figures 3E–H, 1237 neurons counted, 3 mice). In contrast, the Slack channel is expressed in almost all IB4^+^ neurons (Supplementary Figures 3I–L, 968 neurons counted, 3 mice). Consistent with the staining results, patch-clamp recordings showed no difference in large-diameter neuron firing frequency and resting membrane potential between WT and Slack^–/–^ mice (Figures 2A,B,G–I). In contrast, the firing frequencies of the middle/small-diameter neurons significantly increased in Slack^–/–^ mice (Figures 2C–I). These results suggest that Slack channel deletion enhances the excitability of small and middle-sized neurons in the DRG, thus contributing to mechanical hyperalgesia in Slack^–/–^ mice.

**FIGURE 3:**
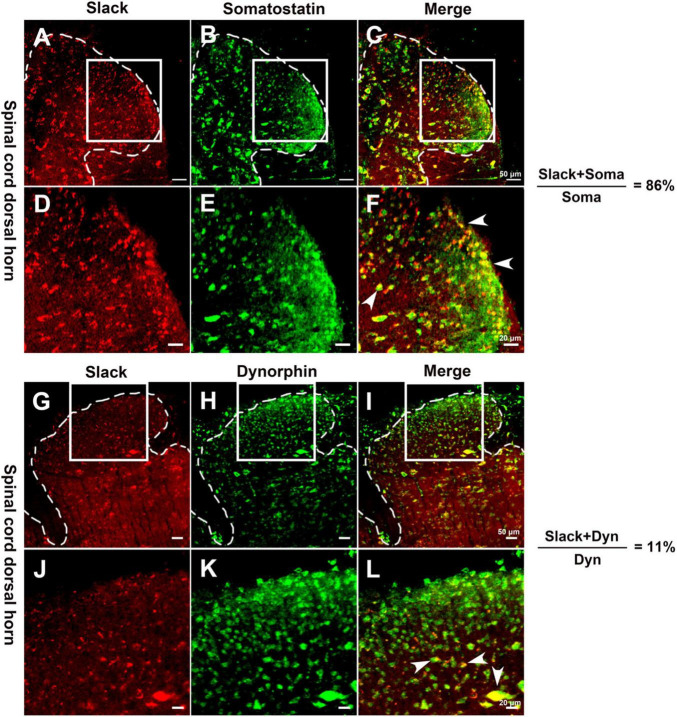
The Slack channel is mainly expressed in Somatostatin positive neurons in the spinal cord. **(A–F)** Double immunofluorescence staining revealed the expression of the Slack channel (**A,D**, in red) in somatostatin positive neurons (**B,E**, in green). The ratio of somatostatin neurons with the Slack channel expression to total somatostatin neurons is 86% (10 sections, 967 neurons counted, 3 mice; **C,F**, in yellow). **(G–L)** Double immunofluorescence staining indicated the absence of Slack channel expression (**G,J**, in red) in dynorphin positive neurons (**H,K**, in green) in the spinal cord. The ratio of dynorphin positive neurons with the expression of Slack channel to total dynorphin positive neurons is 11 % (11 sections, 1123 cells counted, 3 mice; **I,K**, in yellow).

Since the Slack channel expression is also rich in the spinal cord, we further investigated whether the Slack channels in the spinal cord also contribute to mechanical hyperalgesia. We first examined the expression of Slack channels in two neuron sub-populations in the spinal cord related to mechanical pain (Duan et al., 2014). Slack channel expression (in red) was detected in 86% of somatostatin (SOM^+^) positive excitatory neurons (in green) in the superficial dorsal horn of the spinal cord (Figures 3A–F and Supplementary Figure 4A, 10 sections, 967 neurons counted, 3 mice). In contrast, the Slack channel is only expressed in 11% of dynorphin^+^ (Dyn^+^) positive neurons (Figures 3G–L and Supplementary Figure 4B, 11 sections, 1123 neurons, 3 mice). The Slack channel expression in the spinal dorsal horn and SOM expression cannot be detected in the negative control staining without primary antibodies (Supplementary Figures 4C,D). Also, the Slack channel expression cannot be detected in the spinal cord slice of Slack^–/–^ mice (Supplementary Figures 4E–H). These results suggest that the Slack channel is probably involved in mechanical pain by regulating the excitability of SOM^+^ neurons in the spinal cord.

### Slack Channel Deletion Enhances the Excitability of SOM^+^ Neurons in the Spinal Superficial Dorsal Horn

**FIGURE 4:**
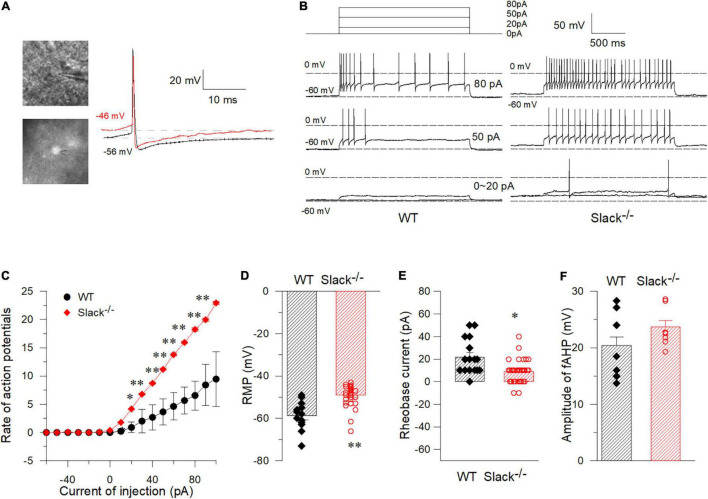
Whole-cell current-clamp recordings in WT and Slack^–/–^ spinal cord somatostatin-positive neurons (SOM^+^ neurons). **(A)** Pictured SOM^+^ neurons in the spinal cord dorsal horn that were labeled by fluorescence and were recorded by patch clamp. Sample spontaneous APs were recorded with I = 0. **(B)** Sample traces of APs elicited in SOM^+^ neurons in the spinal cord of WT-type mice (left) and Slack^–/–^ mice (right) by injected currents as indicated. **(C)** Firing rates of action potentials with different injected currents in spinal cord SOM^+^ neurons in WT mice and Slack^–/–^ mice (Unpaired *T*-test, firing rates from 40 to 120 pA show significant statistical difference, *P* < 0.05). **(D)** The RMP (Resting Membrane Potential) of SOM^+^ neurons in the spinal cord of the WT mice and Slack^–/–^ mice. The averaged RMP of WT mice is –58.8 ± 1.9 mV (WT, *n* = 15, mice *n* = 8, age 11–14 weeks), while the average RMP of Slack^–/–^ mice is –49.08 ± 0.85 mV (Slack^–/–^, *n* = 32, mice *n* = 12; age 11–14 weeks, unpaired *T*-test, *P* < 0.01). **(E)** The averaged rheobase currents that elicit an action potential in the WT mice and Slack null mice were shown (*P* < 0.05). **(F)** No difference of the amplitude of fAHP (fast-afterhyperpolarization) between WT mice and Slack^–/–^ mice. *P < 0.05, ^**^P < 0.01.

**FIGURE 5:**
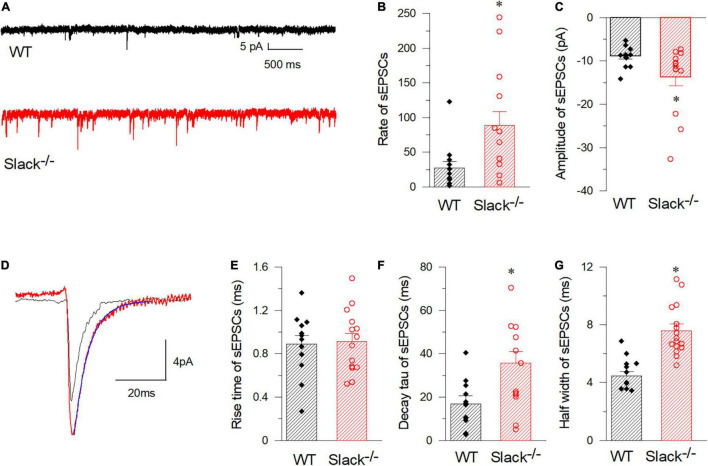
SOM^+^ neurons in the spinal cord of Slack^–/–^ mice exhibit enhanced frequency of spontaneous EPSCs. **(A)** Representative EPSCs of SOM^+^ neurons in WT (black) and Slack^–/–^ (red) mice. **(B)** Averaged spontaneous EPSC frequencies (WT, *n* = 12, 27.2 ± 9.3 times/per min, 3 mice, age 11–13 weeks; Slack^–/–^, *n* = 14, 88.7 ± 19.7 times/per min, 4 mice, age 11–13 weeks; *P* = 0.014). **(C)** Amplitudes of sEPSCs of SOM^+^ neurons in WT and Slack^–/–^ mice (WT, 12 cells, 479 events, –8.9 ± 0.43 pA; Slack^–/–^, 14 cells, 1356 events, –13.7 ± 1.9 pA; *P* = 0.011). **(D)** Typical spontaneous EPSCs from SOM^+^ neurons in WT (black) and Slack^–/–^ (red) mice. **(E)** The averaged activation time course of spontaneous EPSCs from SOM^+^ neurons in WT and Slack^–/–^ mice (WT, *n* = 12, 0.88 ± 0.08 ms; Slack^–/–^, *n* = 14, 0.91 ± 0.08 ms; *P* = 0.82). **(F)** Deactivation time course values of spontaneous EPSCs from SOM^+^ neurons in WT and Slack^–/–^ mice (WT, *n* = 12, 16.9 ± 3.77 ms; Slack^–/–^, *n* = 14, 35.8 ± 5.25 ms; unpaired *T*-test; *P* = 0.037). **(G)** Half width of spontaneous EPSCs from SOM^+^ neurons in WT and Slack^–/–^ mice (WT, *n* = 12, 4.77 ± 0.8 ms; Slack^–/–^, *n* = 14, 7.58 ± 0.8 ms; *P* = 0.001). *p < 0.05.

To test whether the excitability of SOM^+^ neurons in the spinal dorsal horn in Slack^–/–^ mice is altered, we used patch-clamp to measure the activity of SOM^+^ neurons in the spinal dorsal horn of WT and Slack^–/–^ mice. To label SOM^+^ neurons, we crossed SOM^Cre/+^ mice (Taniguchi et al., 2011) with ROSA^Tomato/+^ reporter mice, resulting in double homozygous mice referred to as SOM^+^-Tomato^+^ (Zhu et al., 2014). We then crossed these homogenous SOM^+^-Tomato^+^ mice with Slack^–/–^ mice to obtain SOM^+^-Tomato^+^-Slack^+/–^ mice first, then the SOM^+^-Tomato^+^-Slack^+/–^ mice were crossed and the next generation mice were selected to cross again until we got SOM^+^-Tomato^+^-WT and SOM^+^-Tomato^+^-Slack^–/–^ mice. We next examined the evoked APs of SOM^+^ neurons in the spinal superficial dorsal horn with whole-cell patch-clamp configuration by directly selecting SOM^+^ neurons under a fluorescent microscope (Figure 4A). Our data revealed that the average AP firing rates of SOM^+^ neurons in Slack^–/–^ mice were more robustly elevated than those in WT littermate mice under the same injected current levels (Figures 4B,C). This increase in AP firing rates could be partially attributed to the increased resting membrane potential (RMP), which was not observed in the DRG neurons of Slack^–/–^ mice (Figures 2, 4D). Consequently, the rheobase current that evoked an initial AP also decreased (Figure 4E). However, the average AP amplitude, AP half-width, and fAHP (afterhyperpolarization) amplitudes in WT and Slack^–/–^ SOM^+^ neurons did not significantly differ (Figure 4F and Table 1). Since the previous study has demonstrated heterogeneous populations of SOM^+^ neurons, we thus further categorized the firing patterns and observed whether the deletion of the Slack channel altered the firing patterns. Our results showed the firing rates were increased in both the delayed and tonic firing neurons (Supplementary Figure 5). Meanwhile, the frequency and amplitude of spontaneous EPSCs (s- EPSCs) recorded in SOM^+^ neurons in Slack^–/–^ mice were also robustly elevated compared with WT neurons (Figures 5A–D). Despite the activation time course of s-EPSCs did not show a significant difference between WT and Slack^–/–^ neurons (Figure 5E), the averaged decay time course of s-EPSCs in Slack^–/–^ neurons was longer than that in WT neurons (Figure 5F). In addition, the average half-width of the s-EPSCs in SOM^+^ neurons in the spinal superficial dorsal horn was higher than that in WT neurons (Figure 5G and Table 1). These data indicate that the excitability of Slack^–/–^ SOM^+^ neurons is higher than that of WT SOM^+^ neurons.

**TABLE 1:** Comparison of the electrophysiological properties of SOM^+^-Tomato^+^-WT and SOM^+^-Tomato^+^-Slack^–/–^ neurons in the spinal dorsal horn.

	WT			Slack^–/–^			
	mean	SEM	N (cells)	mean	SEM	N (cells)	*P*-value
Rheobase (pA)	21.86	3.90	15	9.14	1.73	32	0.008
Amp of fAHP (mV)	20.37	1.55	7	23.65	1.18	8	0.19
Amp of sAHP (mV)	2.86	0.74	15	3.48	0.43	32	0.23
RMP (mV)	–58.8	1.88	15	–49.04	0.85	32	0.00029
amplitude of AP (mV)	94.58	1.81	15	92.43	1.84	32	0.30
Half width of AP	1.60	0.13	15	1.71	0.06	32	0.23
−60 pA AP count	0.00	0.00	15	0.01	0.001	32	0.50
−50 pA AP count	0.00	0.00	15	0.00	0.00	32	0.48
−40 pA AP count	0.00	0.00	15	0.00	0.00	32	0.41
−30 pA AP count	0.00	0.00	15	0.00	0.00	32	0.5
−20 pA AP count	0.00	0.00	15	0.01	0.002	32	0.6
−10 pA AP count	0.00	0.00	15	0.06	0.01	32	0.49
0 pA AP count	0.00	0.00	15	0.35	0.02	32	0.45
10 pA AP count	0.25	0.49	15	1.78	0.05	32	0.28
20 pA AP count	0.91	1.09	15	4.15	0.10	32	0.24
30 pA AP count	2.00	2.42	15	6.80	0.15	32	0.13
40 pA AP count	2.68	2.64	15	8.68	0.16	32	0.03
50 pA AP count	3.64	2.70	15	11.18	0.18	32	0.007
60 pA AP count	4.64	2.84	15	13.81	0.23	32	0.007
70 pA AP count	5.63	2.81	15	15.94	0.25	32	0.005
80 pA AP count	6.54	3.03	15	18.23	0.28	32	0.01
90 pA AP count	8.42	4.30	15	19.97	0.30	32	0.005
100 pA AP count	9.46	5.73	15	22.94	0.30	32	0.002
Amp of sEPSCs (pA)	–8.87	0.71	12	–13.72	1.53	14	0.04
Decay tau of sEPSCs (ms)	16.91	3.77	12	35.75	5.25	14	0.006
Half width of sEPSCs (ms)	4.47	0.28	12	7.58	0.49	14	0.001
Rate of sEPSCs (times/min)	27.17	9.26	12	88.66	19.7	14	0.025
Rise time of sEPSCs (ms)	0.91	0.08	11	0.89	0.08	14	0.827

To characterize the molecular basis of the altered excitability of SOM^+^ neurons, we measured Na^+^-dependent potassium currents in WT and Slack^–/–^ mice. In WT SOM^+^ neurons, the K^+^ background current was elicited by 70 mM Na^+^ using −120 mV hyperpolarizing voltage within the first 3–5 min following whole-cell patch-clamp configuration (Supplementary Figure 6A). In contrast, a small background current was observed with 0 mM Na^+^ (Supplementary Figure 6B). The K_Na_ current was blocked by extracellular 20 mM Cs^+^ and restored by washing out extracellular Cs^+^ (Supplementary Figure 6D). There was no Na^+^-dependent current in spinal SOM^+^ neurons in Slack^–/–^ mice (Supplementary Figure 6C), indicating that the Na^+^ dependent current arose from the Slack channel.

### *Kcnt1* Gene Expression in the Spinal Cord Relieves Mechanical Hyperalgesia in Slack^–/–^ Mice

**FIGURE 6:**
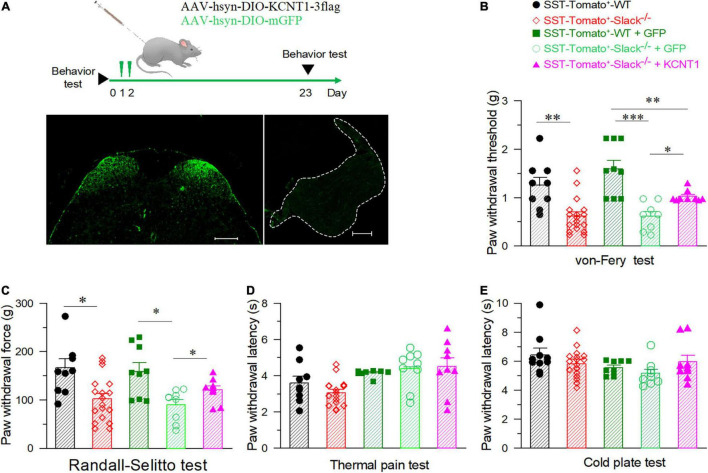
The elevation of mechanical pain thresholds in Slack^–/–^ mice after the compensatory expression of the Slack channel in spinal SOM^+^ neurons. **(A)** Experimental timeline design for adult behavior test (upper). The GFP expression in the SOM^+^ neurons in the spinal cord dorsal horn after ITH (left), no GFP signal in the DRG after ITH (right). **(B)** Mechanical pain thresholds estimated by von Frey tests in different mice groups: SOM^+^-tomato^+^-WT mice (*n* = 9), SOM^+^-tomato^+^-Slack^–/–^ mice (*n* = 18), SOM^+^-tomato^+^-WT + GFP mice (*n* = 9), and SOM^+^-tomato^+^-Slack^–/–^ + KCNT1 mice (*n* = 9). The significant difference of mechanical pain threshold was shown between SOM^+^-tomato^+^-WT and SOM^+^-tomato^+^-Slack^–/–^ mice (One-way ANOVA, *Q*-value = 3.296, *P* = 0.01). The significant difference of mechanical pain thresholds between SOM^+^-tomato^+^-Slack^–/–^ + KCNT1 mice and SOM^+^- tomato^+^- Slack^–/–^ + GFP mice (One-way ANOVA, *Q* = 2.157, *P* = 0.008) or SOM^+^-tomato^+^-WT mice (*Q* = 1.506, *P* = 0.023) were also shown. *P < 0.05, ^**^P < 0.01, ^***^P < 0.001. **(C)** Mechanical pain thresholds were assessed by the Randall-Sellito test in different groups of mice as indicated. Significant difference of mechanical pain threshold was shown between SOM^+^-tomato^+^-WT (*n* = 9) and SOM^+^-tomato^+^-Slack^–/–^ mice (*n* = 18, One-way ANOVA, *P* = 0.014); The significant difference of mechanical pain thresholds between SOM^+^-tomato^+^-WT + GFP mice (*n* = 9) and SOM^+^- tomato^+^- Slack^–/–^ + GFP mice (*n* = 9, *P* = 0.01); between SOM^+^-tomato^+^-Slack^–/–^ + GFP mice (*n* = 9) and SOM^+^- tomato^+^- Slack^–/–^ + KCNT1 mice (*n* = 9, *P* = 0.04) were also shown. **(D,E)** No significant difference of thermal pain **(D)** and cold pain **(E)** sensitivity between SOM^+^-tomato^+^-WT mice and SOM^+^-tomato^+^-Slack^–/–^ mice before and after ITH (unpaired itT-test, *P* = 0.58 and *P* = 0.4, respectively).

**FIGURE 7:**
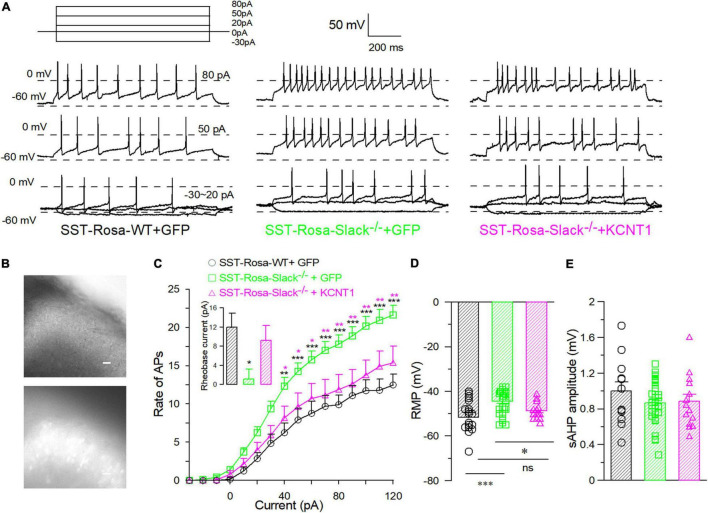
The compensatory expression of the Slack channel in spinal SOM^+^ neurons in the Slack^–/–^ mice removed the over-activity of SOM^+^ neurons. **(A)** Sample action potentials of spinal SOM^+^ neurons (APs) elicited by current injection (–30, 0, 20, 50, and 80 pA) in SOM^+^-tomato^+^-WT mice (left), SOM^+^-tomato^+^-Slack^–/–^ + GFP (middle) and SOM^+^-tomato^+^-Slack^–/–^ + KCNT1 (right) mice, respectively. **(B)** The pictures of patched SOM^+^ neurons selected by fluorescence of tomato^+^ neurons in the spinal slice. **(C)** The action potential firing rates of the spinal SOM^+^ neurons in SOM^+^-tomato^+^-WT, SOM^+^-tomato^+^-Slack^–/–^ + GFP and SOM^+^-tomato^+^-Slack^–/–^ + KCNT1 mice (SOM^+^- tomato^+^-WT, *n* = 18, 4 mice, age 11–14 weeks; SOM^+^-tomato^+^-Slack^–/–^ + GFP, *n* = 34, 4 mice, age 11–14 weeks; SOM^+^-tomato^+^-Slack^–/–^ + KCNT1, *n* = 14, 3 mice, age 11–14 weeks). Rheobase currents showed in small bar chart. The rheobase currents in spinal SOM^+^ neurons in SOM^+^-tomato^+^-WT mice (12 ± 2.9 pA, *n* = 12), SOM^+^-tomato^+^-Slack^–/–^ + GFP mice (1.1 ± 2.0 pA, *n* = 35) and SOM^+^-tomato^+^-Slack^–/–^ + KCNT1 (9.2 ± 3 pA, *n* = 13; one-way ANOVA, *F* = 4.738, *P* = 0.0126. *P < 0.05). **(D)** The averaged RMP of spinal SOM^+^ neurons in SOM^+^-tomato^+^-WT mice (–51.7 ± 2.4 mV, *n* = 18), SOM^+^-tomato^+^-Slack^–/–^ + GFP mice (–44.4 ± 0.9 mV, *n* = 34) and SOM^+^-tomato^+^-Slack^–/–^ + KCNT1 mice (–48.6 ± 1.0 mV, *n* = 14). (One way ANOVA, SOM^+^-tomato^+^-WT+GFP vs. SOM^+^-tomato^+^-Slack^–/–^ + GFP: source of variation: *F* = 8.0, *P* = 0.001; SOM^+^-tomato^+^-Slack^–/–^ + GFP vs. SOM^+^-tomato^+^-Slack^–/–^ + KCNT1, *P* = 0.045. SOM^+^-tomato^+^-WT vs. SOM^+^-tomato^+^-Slack^–/–^ + KCNT1, *P* = 0.3, *P < 0.05). **(E)** The normalized average AHP (afterhyperpolarization) amplitudes of spinal SOM^+^ neurons in three groups did not show significant differences (SOM^+^-Tomato^+^-WT, *n* = 12; SOM^+^-Tomato^+^-Slack^–/–^ + GFP, *n* = 32; SOM^+^-Tomato^+^-Slack^–/–^ + KCNT1, *n* = 13, one-way ANOVA, *F* = 1.644, *P* = 0.2024). *P < 0.05, **P < 0.01, ***P ≦ 0.001.

To further address the role of the Slack channel in spinal SOM^+^ neurons in mechanical pain sensing, we compensated for *Kcnt1* expression in the spinal SOM^+^ neurons of SOM^+^-tomato^+^-Slack^–/–^ mice by intrathecal injection of the AAV-hsyn-DIO-KCNT1-3flag virus. SOM^+^-tomato^+^-Slack^–/–^ mice with intrathecal injection (ITH) of the AAV-hsyn-DIO-GFP virus were used as control. Effective *Kcnt1* expression in SOM^+^ neurons was confirmed by Western Blot and electrophysiology recording, while GFP expression was observed by fluorescence microscopy in the spinal cord of mice in the control group (Supplementary Figures 6E–G and Figure 6A left). The DRG was not infected and did not show the expression of the GFP in mice of the control group at the titrate concentration we used (Figure 6A right). Next, we measured the mechanical pain thresholds of these mice using the von Frey test and paw withdrawal forces using the Randall-Selitto test. Before ITH, SOM^+^-Tomato^+^-Slack^–/–^ mice exhibited mechanical hyperalgesia (Figures 6B,C) without altered sensitivity to thermal and cold pain, similar to Slack^–/–^ mice (Figures 6D,E). After compensatory Slack channel expression in the spinal SOM^+^ neurons, SOM^+^-Tomato^+^-Slack^–/–^ mice mechanical pain threshold was significantly higher than the threshold of GFP-expressing SOM^+^-tomato^+^-Slack^–/–^ control mice in the von Frey test, but the mice still show somewhat mechanical hyperalgesia compared with SOM^+^-Tomato^+^-WT mice with GFP expression (Figure 6B). Meanwhile, the compensatory Slack channel expressed SOM^+^-Tomato^+^-Slack^–/–^ mice did not exhibit mechanical hyperalgesia in the Randall-Selitto test compared with SOM^+^-Tomato^+^-WT mice, while the GFP-expressing SOM^+^-tomato^+^-Slack^–/–^ control group mice still manifest mechanical hyperalgesia (Figure 6C). Meanwhile, virus injection of GFP in WT mice or *Kcnt1* in Slack^–/–^ mice did not alter sensitivity to thermal and cold pain (Figures 6D,E). Furthermore, consistent with the results of behavioral tests, patch-clamp recordings indicated that compensating *Kcnt1* gene expression in spinal SOM^+^ neurons decreased their AP firing rates (Figures 7A–C) and restored the Na^+^-dependent current (Supplementary Figure 6). However, the enhanced RMP was not completely restored (Figure 7D and Table 2), and small AHP amplitudes in spinal SOM^+^ neurons were also not altered by Slack re-expression (Figure 7E and Table 2). Taken together, we conclude that the Slack channel in SOM^+^ neurons is also involved in mechanical pain-sensing.

**TABLE 2:** Comparison of electrophysiological properties of SOM^+^-Tomato^+^-WT + GFP, SOM^+^-Tomato^+^-Slack^–/–^+ GFP, and SOM^+^-Tomato^+^-Slack^–/–^ + KCNT1 neurons in the spinal dorsal horn.

	WT			Slack^–/–^+GFP			Slack^–/–^+KCNT1		
	mean	SEM	*n*	mean	SEM	*n*	mean	SEM	*n*
Rheobase (pA)	12.00	2.91	12	1.14	2.04	35	9.23	3.09	13
Normalized AHP amplitude	1.00	0.10	12	0.87	0.04	32	0.88	0.08	13
RMP (mV)	–51.72	1.31	18	–44.38	0.95	34	–48.64	1.04	14
−20 pA AP count	0	0	12	0.11	0.06	35	0	0	13
−10 pA AP count	0.02	0.02	12	0.44	0.18	35	0.08	0.08	13
0 pA AP count	0.21	0.16	12	1.43	0.35	35	0.73	0.35	13
10 pA AP count	1.27	0.46	12	3.75	0.54	35	2.10	0.58	13
20 pA AP count	2.87	0.78	12	6.25	0.78	35	4.13	0.84	13
30 pA AP count	4.88	1.18	12	9.38	0.98	35	6.04	1.05	13
40 pA AP count	6.28	1.23	12	12.34	1.11	35	8.51	1.28	13
50 pA AP count	7.92	1.50	12	14.27	1.10	35	10.25	1.59	13
60 pA AP count	8.81	1.55	12	15.76	1.17	35	11.68	1.72	13
70 pA AP count	9.69	1.39	12	17.02	1.14	35	12.06	1.84	13
80 pA AP count	9.91	1.24	12	17.82	1.19	35	13.06	1.83	13
90 pA AP count	11.08	1.31	12	18.89	1.21	35	13.66	1.76	13
100 pA AP count	11.76	1.39	12	20.17	1.28	35	14.91	1.80	13
110 pA AP count	11.81	1.44	12	20.94	1.38	35	15.85	1.78	13
120 pA AP count	12.50	1.39	12	21.64	1.25	35	16.28	1.87	13

*PWL, Paw Withdrawal Latency; AHP, Afterhyperpolarization; RMP, Resting Membrane Potential; AP, Action Potential; s-EPSCs, Spontaneous Excitatory Postsynaptic Current.*

### Conditional Knockout of the *Kcnt1* Gene in Spinal SOM^+^ Neurons Caused Mechanical Pain Hypersensitivity of Mice

**FIGURE 8:**
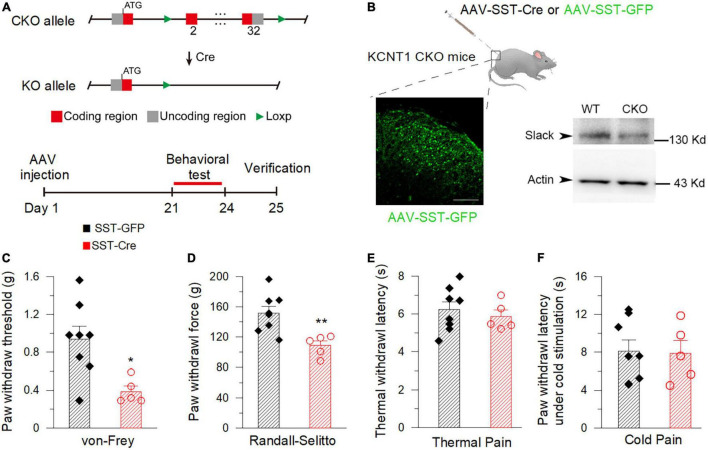
Deletion of the Slack channel in the spinal SOM^+^ neurons decreases the mechanical pain threshold in mice. **(A)** The AAV-Cre injection-based conditional knockout (CKO) strategy of the *Kcnt1* gene. **(B)** Successful expression of AAV-SST-GFP verified by green fluorescence detected in the spinal dorsal horn in mice (left, Scale bar, 100 μm). The down-regulation of Slack protein expression in the spinal dorsal horn of CKO mice 3 weeks after AAV-SST-Cre injection is verified by Western Blot (right, two-tail unpaired *T*-test: *P* < 0.05, *n* = 4). **(C)** Mechanical pain thresholds of the AAV-SST-GFP injected mice (0.93 ± 0.14 g, *n* = 8) and the AAV-SST-Cre injected mice (0.39 ± 0.058 g, *n* = 5). The * shows statistically significant difference (von Frey test, student *T*-test: *P* = 0.0171). **(D)** Mechanical pain thresholds of the AAV-SST-GFP injection mice (151.70 ± 9.05 g, *n* = 8) and the AAV-SST-Cre injection mice (108.80 ± 6.18 g, *n* = 5) in Randall-Selitto test. **Shows a statistically significant difference (Two-tailed *T*-test: *P* = 0.0058). **(E,F)** There is no statistically significant difference between the Slack CKO mice and WT mice in thermal pain (**E**, 40% light intensity, AAV-SST-GFP injected: 6.23 ± 0.41 s, *n* = 8, AAV-SST-Cre injected: 5.87 ± 0.34 s, *n* = 5) and cold pain sensitivity (**F**, AAV-SST-GFP: 8.12 ± 1.16 s, *n* = 8, AAV-SST-Cre: 7.89 ± 1.34 s, *n* = 5). (Student *T*-test: *P* > 0.05).

To further address the contribution of Slack channels in the spinal SOM^+^ neurons to mechanical hyperalgesia, the pain behaviors of mice with specific deletion of the Slack channel in SOM^+^ neurons were observed. The mice line with the *Kcnt1* gene flanked by LoxP-site was generated according to the design shown in Figure 8A. The excision of the loxp-flanked Slack channel sequence in spinal SOM^+^ neurons was realized by intrathecal injection of the AAV-SST-Cre virus. The AAV-SST-GFP virus was injected into mice in the control group (Figure 8B). Three weeks later, the virus expression was detected by the observed green GFP fluorescence. The effective Slack channel deletion in SOM^+^ neurons was confirmed by Western Blot (Figure 8B, right). We found Slack channel deletion in spinal SOM^+^ neurons was sufficient to result in mechanical hyperalgesia in mice in both von-Frey and Randall-Selitto tests (Figures 8C,D) but not in thermal and cold pain tests (Figures 8E,F). These data proved the role of the Slack channel in SOM^+^ neurons for maintaining normal mechanical pain sensing in mice.

## Discussion

Our present results purport that the Slack channel deletion causes mechanical hyperalgesia in mice by increasing the excitability of both small neurons in the DRG and SOM^+^ neurons in the spinal cord but does not influence thermal and cold pain sensing. These results are partially consistent with previous studies that reported that thermal pain and cold pain sensitivity were not altered in other lines of Slack channel knockout mice (Lu et al., 2015; Martinez-Espinosa et al., 2015). However, our finding that Slack^–/–^ mice are mechanical hyperalgesia is inconsistent with a previous study that reported no change in mechanical pain in another line of knockout mice (Lu et al., 2015). The major difference between our experiments and the previous study may arise from the experimental method used. In our study, the Randall-Selitto test was used to measure the pressure force that led to hind paw withdrawal in the naive mice following the standard protocol (Anseloni et al., 2003). However, the Randall-Selitto test was used to examine the mechanical pain by measuring tail withdrawal in the previous study, for which the equipment they used was not designed (Paw Pressure Randall Selitto Instrument, IITC Life Science) (Lu et al., 2015). Also, another recent study from the same group used the von Frey test to measure mechanical pain sensitivity in the parsed nerve injury (SNI) model mice and naïve mice, which showed no different mechanical pain sensitivity in naive WT and Slack^–/–^ mice without using the Randall Selitto test (Lu et al., 2021). Thus, we believe in the soundness of the enhanced response to nociceptive mechanical stimuli in Slack^–/–^ mice. Furthermore, nociceptive mechanical stimuli are transduced in capsaicin-insensitive A-fiber (e.g., A-M and A-MH type I) nociceptors, and C-fiber receptors (peripheral sensory neurons) (Dubin and Patapoutian, 2010). IB4^+^ neurons in the DRG are exclusively unmyelinated C-fiber nociceptors that perceive nociceptive mechanical stimuli and inflammatory pain but not thermal and neuropathic pain (Vulchanova et al., 2001; Fang et al., 2006; Abrahamsen et al., 2008), whereas CGRP-positive neurons are thinly myelinated Aδ fiber nociceptors involved in mechanical, thermal/cold and inflammatory pain perception (Pogatzki et al., 2002; Jankowski and Koerber, 2010; Ishida et al., 2014; Melo-Carrillo et al., 2017). Our immunochemistry staining results suggest that the Slack channel is expressed in almost all IB4^+^ neurons and partially expressed in CGRP neurons, also reported in previous studies (Lu et al., 2015; Martinez-Espinosa et al., 2015), and are consistent with the alteration of mechanical pain sensitivity in Slack^–/–^ mice. In addition, Slack channel deletion enhances the excitability of small-diameter DRG neurons, which supports the view that the Slack channel is involved in mechanical pain transduction. Although we cannot exclude the possibility that the Slack channel is also involved in neuropathic pain transduction, the role of Slack channel in neuropathic pain needs further investigation and careful exclusion of the influence of mechanical hyperalgesia.

**FIGURE 9:**
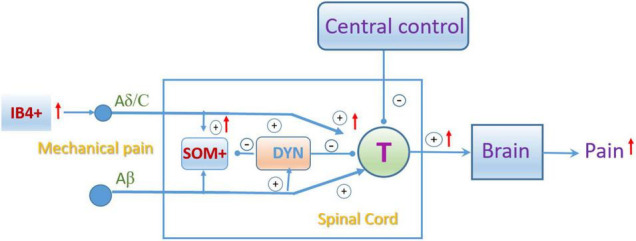
Schematic showing the pain gating control modification by the Slack channel knockout. “IB4^+^,” “SOM^+^,” and “Dyn^+^” represent the isolectin-B4, somatostatin, and dynorphin positive neurons, respectively. “T”: spinal pain signal transmission neuron. “+” and “–”represent excitatory and inhibitory roles in pain transmission, respectively. The red arrow indicated the enhanced activity due to the Slack channel knockout.

In the spinal cord, our X-gal staining showed the rich Slack channel expression in the spinal dorsal horn and scattered expression in other parts of the spinal cord, which is different from previous studies that reported the Slack expression in IB4-positive central terminals in the dorsal horn of the spinal cord. Although the LacZ reporter gene expression is under the control of regulatory elements of the KCNT1 gene transcription, sometimes the indigo product tends to diffuse to surrounding tissues and fade (probably also diffusion in the staining in the DRG) (Trifonov et al., 2016). But the deep indigo staining in the dorsal horn and other parts of the spinal cord cannot be explained as indigo products diffusion and fade. Consistently, the *in situ* hybridization results show a similar mRNA expression pattern of the Slack channel. Although the sense RNA probe also detected some positive signals in the spinal cord, it could be explained as the antisense lncRNA of the Slack channel may exist (Statello et al., 2021). Furthermore, the expression of the Slack protein detected in the spinal cord of the WT mice but not in Slack^–/–^ mice further proves the Slack channel expression in the cell bodies of the spinal cord neurons (Figure 3 and Supplementary Figures 2, 4). The Slack channel expression pattern in the spinal cord was further studied by double immunofluorescence. The co-localization of Slack channel expression with the SOM^+^ neuron in the superficial dorsal horn (mainly in lamina II) was observed whereas only a small amount of colocalization of the Slack channel with the DYN^+^ neurons (mainly in lamina III-V). The absence of staining when the SOM^+^ or DYN^+^ primary antibody was omitted in the control spinal sections proved the specificity of the staining (Supplementary Figure 4). The SOM^+^ and DYN^+^ neurons distribution pattern in the spinal cord we detected is consistent with previously reported results (Huang et al., 2018; Serafin et al., 2021). SOM^+^ neurons located mainly in lamina II (also scattered in III-V lamina) transmit noxious mechanical signals to lamina I projection neurons in the dorsal horn, whereas Dyn^+^ neurons in the outer layer of lamina II (also scattered in I and III-V lamina) serve as inhibitory interneurons to prevent low-threshold mechanical stimuli from activating SOM^+^ neurons (Duan et al., 2014). SOM^+^ neurons and Dyn^+^ neurons are specifically required for mechanical pain transmission rather than the heat and cold noxious pain. The Slack channel is expressed in 86% of SOM^+^ neurons but is only in 11% of DYN^+^ neurons in the spinal cord. Since ablation of the Slack channel and its compensated expression in SOM^+^ neurons altered the SOM^+^ cell excitability indicates that the Slack channel plays an essential role in regulating the activity of SOM^+^ neurons. The gating control theory of pain postulates that pain transmission neurons (T) receive both nociceptive painful input (Aδ/C) and non-painful input (Aβ), whereas the transmissions from Aβ fibers prevent pain sensation information traveling from Aδ/C fibers to the brain. In the spinal cord, Dyn^+^ neurons serve as non-painful inhibitory interneurons preventing mechanical pain transmission from Aδ/C fibers. In Slack^–/–^ mice, both overactivity of IB4^+^ and SOM^+^ neurons enhanced mechanical pain transmission signals resulting in an imbalance of mechanical pain transmission with enhanced pain signals transmitted to the brain (Figure 9). Therefore, the expression of the Slack channel in these neurons represents an inherent inhibition mechanism in the mechanical pain transmission pathway. Thus, our study provides new insights into the role of the Slack channel in the regulation of pain-related circuits.

We also characterized the electrophysiological properties of SOM^+^ neurons in Slack^–/–^ mice. In addition to the enhanced firing rates, the average resting membrane potential of SOM^+^ neurons in Slack^–/–^ mice was also significantly increased, which was not observed in our recording in small-diameter neurons in the DRG but was also observed in IB4^+^ positive DRG neurons in a previous study (Martinez-Espinosa et al., 2015). Accordingly, the measured Na^+^-dependent currents in the SOM^+^ neurons were also comparable to the previous work recorded in IB4^+^ DRG neurons (Martinez-Espinosa et al., 2015). Thus, we speculate that the mechanism of higher Slack channel activation in SOM^+^ neurons either attributed to the rich expression of the Slack channel or attributed to a high local cytosolic Na^+^ concentration close to the Slack channel on the cell membrane in SOM^+^ neurons, which implies that the leaky Na^+^ channel may be expressed in spinal SOM^+^ neurons because the Slack channel needs a high Na^+^ concentration for activation. In the meantime, the enhanced amplitude and firing frequency of sEPSC in the SOM^+^ neurons in Slack^–/–^ mice suggest that deletion of the Slack channel facilitates excitatory transmission. The increased frequency of sEPSC probably reflects the deletion of the Slack channel potentiates excitatory synaptic transmission through a presynaptic mechanism and by increasing the probability of glutamate release at a synapse, which is consistent with the enhanced firing frequency in IB4^+^ DRG neurons. Whether the increased amplitude of sEPSC is explained as presynaptic mechanisms or postsynaptic mechanisms cannot be determined based on current data. The mEPSC recording with an application of TTX may help certify this mechanism. But considering the Slack channel is a sodium-dependent channel, this TTX application may also alter the function of the Slack channel. Further study using genetic and pharmacological manipulation in SOM^+^ neurons or paired recording is required to further address the mechanism. This study also did not test whether the inhibitory postsynaptic current (IPSC) of SOM^+^ neurons is altered or not partially because of an absence of information and labeling of the inhibitory neurons that mediate SOM^+^ neurons. Future work is needed to further dissect the role of the Slack channel in the regulation of IPSP and IPSC of the SOM^+^ neurons.

In addition, spinal SOM^+^ neurons are also upstream of DYN^+^ neurons and contribute to chemical itch transmission, while Slack^–/–^ mice are more sensitive to chemical itch (Martinez-Espinosa et al., 2015; Huang et al., 2018; Fatima et al., 2019), which suggests a possible role of the Slack channel in SOM^+^ neurons in chemical itch transmission. However, these speculations need further investigation.

## Data Availability Statement

The original contributions presented in the study are included in the article/Supplementary Material, further inquiries can be directed to the corresponding author/s.

## Ethics Statement

The animal study was reviewed and approved by the Institutional Animal Care and Use Committee of the Xuzhou Medical University.

## Author Contributions

YL performed ITH injection, electrophysiology recording of spinal cord slice, and pain behavior test on ITH injected mice. Fa-FZ performed all the immunostaining. RW performed the pain behavior test of the Slack^–/–^ mice. YS performed patch-clamp recording on the DRG neurons. QG, Z-SS, Fe-FZ, QZ, D-YZ, and X-HW contributed to data analysis and genotyping of mice lines. Q-YT and ZZ supervised all experiments. YL and ZZ wrote the manuscript. All authors contributed to the article and approved the submitted version.

## Conflict of Interest

The authors declare that the research was conducted in the absence of any commercial or financial relationships that could be construed as a potential conflict of interest.

## Publisher’s Note

All claims expressed in this article are solely those of the authors and do not necessarily represent those of their affiliated organizations, or those of the publisher, the editors and the reviewers. Any product that may be evaluated in this article, or claim that may be made by its manufacturer, is not guaranteed or endorsed by the publisher.

## Supplementary Material

Supplementary Figure 1 Decreased mechanical pain thresholds in female Slack channel Knockout mice. **(A,B)** Mechanical pain thresholds in WT and Slack^–/–^ female mice in von Frey test and Randall-Selitto test. Von Frey test (**A**, WT: 0.96 ± 0.043 g, *n* = 12, Slack^–/–^: 0.65 ± 0.06 g, *n* = 14, Two-tailed *T*-test: *P* = 0.0003) and Randall-Selitto test (**B**, WT: 116.28 ± 16.47 g, *n* = 7, Slack^–/–^: 80.95 ± 4.65 g, *n* = 10, student *T*-test: *P* = 0.0114). **(C,D)** Thermal pain and cold pain sensitivity of WT and Slack^–/–^ female mice. The Paw withdrawal latencies of thermal pain test (**C**, 30% light intensity, WT: 12.96 ± 1.30 s, *n* = 7, Slack^–/–^: 13.20 ± 1.06 s, *n* = 10, *P* = 0.89) and cold pain test (**D**, WT: 6.32 ± 0.41 s, *n* = 7, Slack^–/–^: 6.80 ± 0.54 s, *n* = 10, *P* = 0.49) of Slack^–/–^ mice were not significantly different from the latencies of WT mice. **P* < 0.05, ^**^*P* < 0.05.

Supplementary Figure 2 X-gal staining showing the Slack channel expression patterns in the DRG and spinal cord in mice. **(A)** The expression pattern of the Slack channel in the DRG labeled by X-gal staining in Slack null mice. Scale bar, 100 μm. **(B)** The negative control staining in the DRG neurons of the WT Slack channel. **(C)** The picture of X-gal staining shows the Slack channel expression pattern in the spinal cord. **(D)** The negative control of X-gal staining in the spinal cord in WT mice. **(E)** *In situ* hybridization showing the expression of the Slack channel in the spinal cord neurons in mice. **(F)** The negative control of the *in situ* hybridization with the Slack channel sense probe in the spinal cord. **(G,H)** The immunostaining of the Slack channel in the dorsal horn of the spinal cord in WT-type mice and the Slack^–/–^ mice, respectively.

Supplementary Figure 3 Double immunostaining shows that the Slack channel is expressed in IB4 positive neurons in the DRG. **(A–D)** Double immunofluorescence staining showed almost no colocalization (**C**, Merge) of the Slack channel (**A**, in red) with NF200 positive neurons (**B**, in green) (<1%). **(E–H)** Double immunofluorescent staining shows partially co-localization (**G**, Merge) of the KCNT1 channel (**E**, in red) with calcitonin gene-related peptide (CGRP) (**F**, in green). Mouse number: 3, counted neurons: 1237. **(I–L)** Double immunofluorescent staining shows partial colocalization (**K**, Merge) of the Slack channel (**I**, in red) with isolectin B4-binding protein (IB4) (**J**, in green). Mouse number = 3, counted neurons: 968.

Supplementary Figure 4 The comparison of the expression of the Slack channel in SOM/DYN positive neurons in the spinal cord of the WT-type mice and the Slack channel KO mice. **(A,B)** Double immunofluorescence staining revealed that the expression of the Slack channel (**A**, in red) in somatostatin (in green) positive neurons (**C**, merged in yellow) in the wild type mice but not in dynorphin positive neurons. **(C,D)** The control staining indicates the failure of detection of the Slack channel expression **(C)** or SOM expression without adding the primary antibody in the WT mice. **(E–H)** The failure of detection of the Slack channel expression **(E,G)** in SOM^+^ positive neurons **(F)** and DYN^+^ neurons in the Slack^–/–^ mice.

Supplementary Figure 5 Categorized analysis of action potentials of the SOM^+^ positive neurons in the spinal cord of the WT mice and the Slack^–/–^ mice. **(A)** Firing rates of the tonic action potentials with different injected currents in spinal cord SOM^+^ neurons in WT mice (*n* = 10) and Slack^–/–^ mice (*n* = 12) (Unpaired *T*-test, firing rates from 60 to 100 pA show a significant statistical difference, *P* < 0.05). **(B)** Sample traces of tonic APs elicited in SOM^+^ neurons in the spinal cord of WT-type mice (top, black) and Slack^–/–^ mice (bottom, red) by injected currents as indicated. **(C)** Firing rates of the delayed action potentials with different injected currents in spinal cord SOM^+^ neurons in WT mice (*n* = 6) and Slack^–/–^ mice (*n* = 8) (Unpaired *T*-test, firing rates from 60 to 100 pA show significant statistical difference, *P* < 0.05). **(D)** Sample traces of delayed APs elicited in SOM^+^ neurons in the spinal cord of WT-type mice (top, black) and Slack^–/–^ mice (bottom, red) by injected currents as indicated. **P* < 0.05, ***P* < 0.01.

Supplementary Figure 6 The spinal SOM^+^ neurons in the Slack^–/–^ mice lost sodium-dependent potassium currents. **(A)** The sample traces of the sodium-dependent potassium current were recorded in the spinal SOM^+^ neurons in WT mice with 70 mM cytosolic sodium (black). The sodium-dependent currents were inhibited by 20 mM CsCl (blue) and could be restored by washing out the CsCl (pink). **(B,C)** The sodium-dependent currents were not observed in 0 mM cytosolic sodium in spinal SOM^+^ neurons or the Slack^–/–^ mice with 70 mM cytosolic Na^+^. **(D)** The averaged Slack channel current levels in the condition of 70 mM cytosolic sodium or 0 mM sodium, 20 mM CsCl, and washing out of CsCl in the spinal SOM^+^ neurons in the WT mice and Slack^–/–^ mice, respectively. **(E)** Western Blot showed compensated expression of the Slack channel in the spinal cord of Slack^–/–^ mice. **(F)** The sodium-dependent currents were measured after compensated expression of the Slack channel in the SOM^+^ neurons in the spinal cord of Slack^–/–^ mice. **(G)** The average magnitudes of the sodium dependent currents in **(F)**.
