# Supplementary figures and images for: The Slack Channel Deletion Causes Mechanical Pain Hypersensitivity in Mice
Source: Front Mol Neurosci. 2022 Mar 11;15:811441. doi: 10.3389/fnmol.2022.811441 (PMC8963359; doi:10.3389/fnmol.2022.811441)

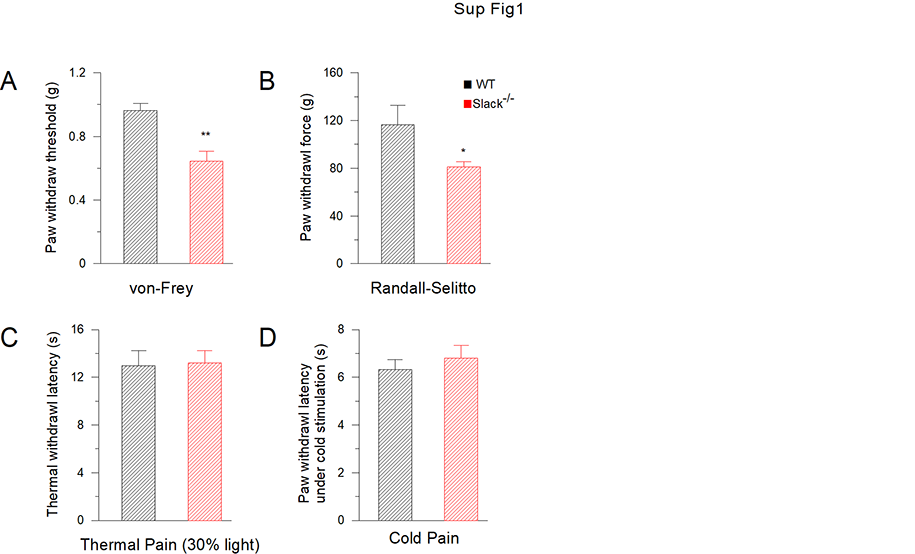

Supplement: Supplementary Figure 1 — Decreased mechanical pain thresholds in female Slack channel Knockout mice. (A,B) Mechanical pain thresholds in WT and Slack–/– female mice in von Frey test and Randall-Selitto test. Von Frey test (A, WT: 0.96 ± 0.043 g, n = 12, Slack–/–: 0.65 ± 0.06 g, n = 14, Two-tailed T-test: P = 0.0003) and Randall-Selitto test (B, WT: 116.28 ± 16.47 g, n = 7, Slack–/–: 80.95 ± 4.65 g, n = 10, student T-test: P = 0.0114). (C,D) Thermal pain and cold pain sensitivity of WT and Slack–/– female mice. The Paw withdrawal latencies of thermal pain test (C, 30% light intensity, WT: 12.96 ± 1.30 s, n = 7, Slack–/–: 13.20 ± 1.06 s, n = 10, P = 0.89) and cold pain test (D, WT: 6.32 ± 0.41 s, n = 7, Slack–/–: 6.80 ± 0.54 s, n = 10, P = 0.49) of Slack–/– mice were not significantly different from the latencies of WT mice. *P < 0.05, **P < 0.05. [file Image_1.TIF]

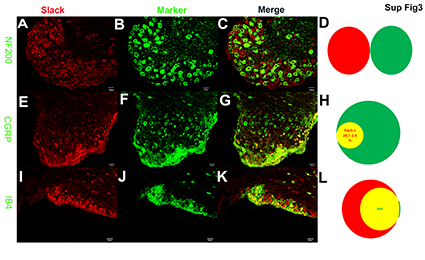

Supplement: Supplementary Figure 3 — Double immunostaining shows that the Slack channel is expressed in IB4 positive neurons in the DRG. (A–D) Double immunofluorescence staining showed almost no colocalization (C, Merge) of the Slack channel (A, in red) with NF200 positive neurons (B, in green) (<1%). (E–H) Double immunofluorescent staining shows partially co-localization (G, Merge) of the KCNT1 channel (E, in red) with calcitonin gene-related peptide (CGRP) (F, in green). Mouse number: 3, counted neurons: 1237. (I–L) Double immunofluorescent staining shows partial colocalization (K, Merge) of the Slack channel (I, in red) with isolectin B4-binding protein (IB4) (J, in green). Mouse number = 3, counted neurons: 968. [file Image_3.TIF]

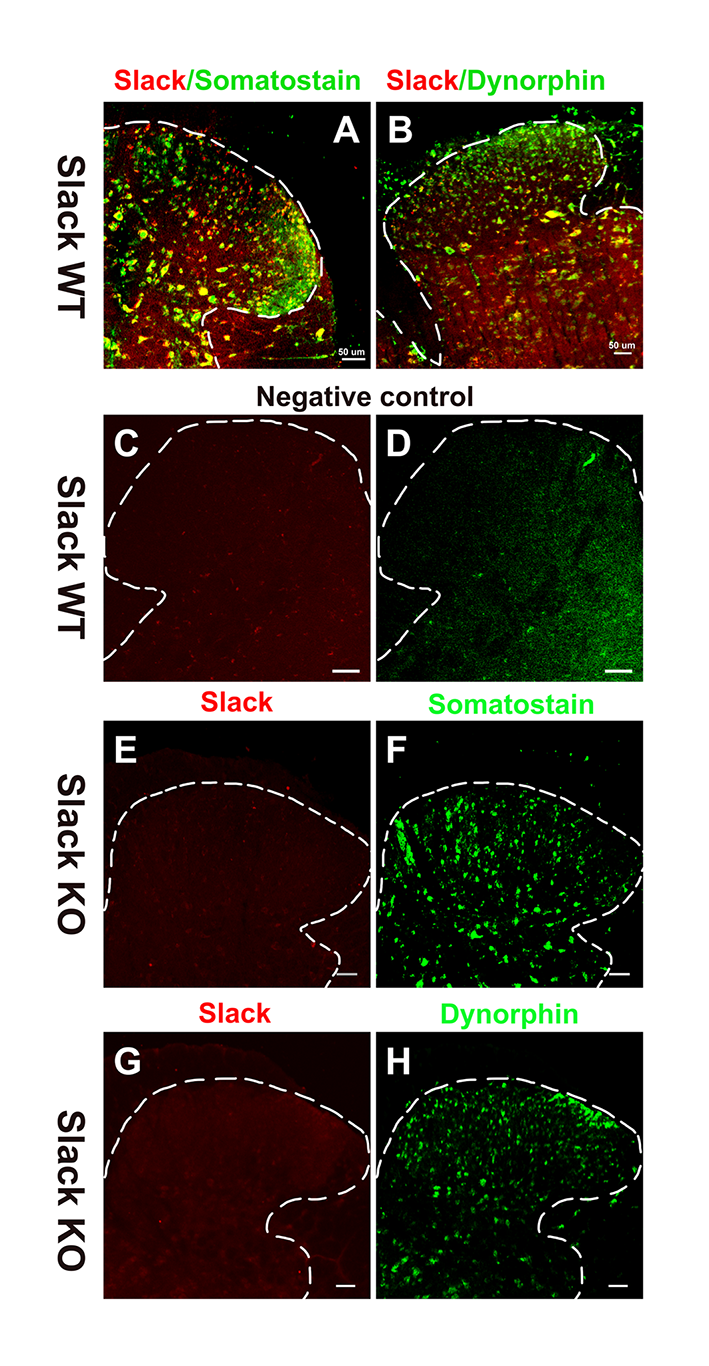

Supplement: Supplementary Figure 4 — The comparison of the expression of the Slack channel in SOM/DYN positive neurons in the spinal cord of the WT-type mice and the Slack channel KO mice. (A,B) Double immunofluorescence staining revealed that the expression of the Slack channel (A, in red) in somatostatin (in green) positive neurons (C, merged in yellow) in the wild type mice but not in dynorphin positive neurons. (C,D) The control staining indicates the failure of detection of the Slack channel expression (C) or SOM expression without adding the primary antibody in the WT mice. (E–H) The failure of detection of the Slack channel expression (E,G) in SOM+ positive neurons (F) and DYN+ neurons in the Slack–/– mice. [file Image_4.TIF]

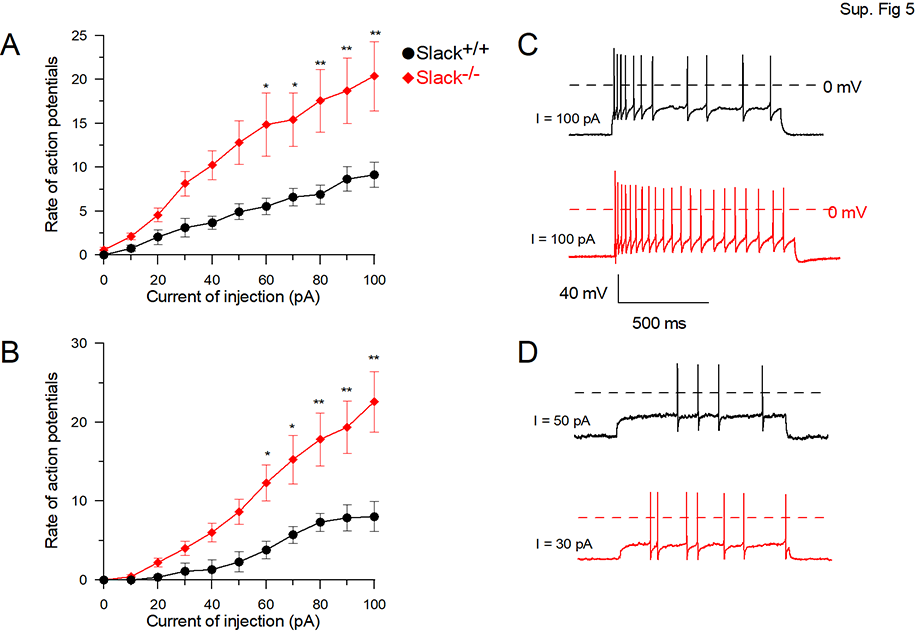

Supplement: Supplementary Figure 5 — Categorized analysis of action potentials of the SOM+ positive neurons in the spinal cord of the WT mice and the Slack–/– mice. (A) Firing rates of the tonic action potentials with different injected currents in spinal cord SOM+ neurons in WT mice (n = 10) and Slack–/– mice (n = 12) (Unpaired T-test, firing rates from 60 to 100 pA show a significant statistical difference, P < 0.05). (B) Sample traces of tonic APs elicited in SOM+ neurons in the spinal cord of WT-type mice (top, black) and Slack–/– mice (bottom, red) by injected currents as indicated. (C) Firing rates of the delayed action potentials with different injected currents in spinal cord SOM+ neurons in WT mice (n = 6) and Slack–/– mice (n = 8) (Unpaired T-test, firing rates from 60 to 100 pA show significant statistical difference, P < 0.05). (D) Sample traces of delayed APs elicited in SOM+ neurons in the spinal cord of WT-type mice (top, black) and Slack–/– mice (bottom, red) by injected currents as indicated. *P < 0.05, **P < 0.01. [file Image_5.TIF]

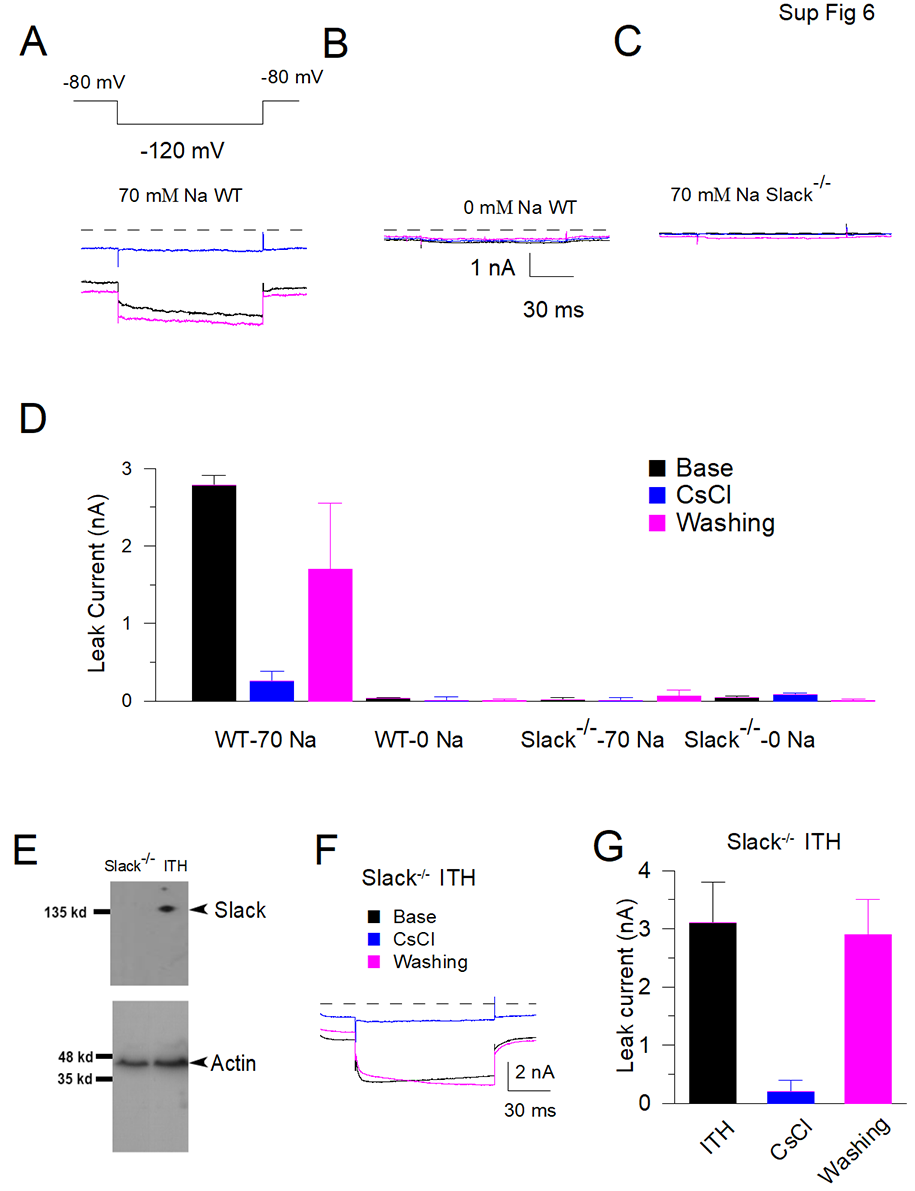

Supplement: Supplementary Figure 6 — The spinal SOM+ neurons in the Slack–/– mice lost sodium-dependent potassium currents. (A) The sample traces of the sodium-dependent potassium current were recorded in the spinal SOM+ neurons in WT mice with 70 mM cytosolic sodium (black). The sodium-dependent currents were inhibited by 20 mM CsCl (blue) and could be restored by washing out the CsCl (pink). (B,C) The sodium-dependent currents were not observed in 0 mM cytosolic sodium in spinal SOM+ neurons or the Slack–/– mice with 70 mM cytosolic Na+. (D) The averaged Slack channel current levels in the condition of 70 mM cytosolic sodium or 0 mM sodium, 20 mM CsCl, and washing out of CsCl in the spinal SOM+ neurons in the WT mice and Slack–/– mice, respectively. (E) Western Blot showed compensated expression of the Slack channel in the spinal cord of Slack–/– mice. (F) The sodium-dependent currents were measured after compensated expression of the Slack channel in the SOM+ neurons in the spinal cord of Slack–/– mice. (G) The average magnitudes of the sodium dependent currents in (F). [file Image_6.tif]
